# Exploring Parent Support Needs During and After Adolescent Suicide Crisis Emergency Department Visits: A Qualitative Investigation

**DOI:** 10.1111/hex.70775

**Published:** 2026-07-29

**Authors:** Demee Rheinberger, Katherine Boydell, Susanne Oliver Armstrong, Sally Ann Pollard, Julia Lessing, Lauren McGillivray, Fiona Shand

**Affiliations:** ^1^ Black Dog Institute University of New South Wales, Hospital Road Randwick Australia; ^2^ Tyree Foundation Institute of Health Engineering University of New South Wales Sydney Australia

**Keywords:** adolescent, emergency care, parents, qualitative research, suicide

## Abstract

**Background:**

Adolescents are the age cohort most likely to attend an emergency department (ED) for suicide crisis, often accompanied by a parent. Parents report feeling as though they are not appropriately supported to provide the critical life sustaining care their adolescent child needs after discharge from the ED. This study sought to understand what support parents need during and after the ED presentation.

**Methods:**

Semi‐structured, online interviews were conducted with 20 parents of adolescents (12–18 years) who had attended an Australian ED for suicide crisis.

**Results:**

Reflexive thematic analysis indicated there were three domains of support parents' desired: (1) information about, and active involvement in, the emergency department care, (2) information about how to keep their adolescent child safe and support recovery, and (3) tools to support parents' own wellbeing.

**Conclusion:**

These findings provide a preliminary indication of the support parents need and what resources may need to be developed to educate and support parents, which could be integrated into the ED procedures. Improving the way in which parents are supported to care for their adolescent child may help reduce recurrent adolescent suicide attempts and improve parental wellbeing so they can more effectively care for their adolescent child.

**Patient or Public Contribution:**

Parents who had lived experience of attending the ED with their suicidal adolescent were involved in this study as peer researchers. These peer researchers helped devise the interview guide, conducted interviews with participants, and contributed to the analysis and the writing of this paper.

## 1 Introduction

Adolescents are the age cohort most likely to present to an emergency department (ED) during a suicide crisis (suicidal thoughts, feelings, plans or behaviours), in many developed countries, including Australia [1], and are also the age group most often hospitalised in Australia for suicide crisis [2]. Parents play a vital role in assisting their adolescent child to access crisis care such as EDs and ongoing mental healthcare in the community [3]. However, parents often experience their own struggles during this time, including heightened stress, anxiety, fear and depression [4]. Parents who are psychologically distressed are less able to make effective decisions about their child's care [5], suggesting parents are possibly struggling to provide appropriate care to their suicidal adolescent at a time when it is most critical.

Numerous studies have demonstrated the benefits of providing education and support to parents of suicidal adolescents. For instance, several studies have shown that parents respond positively to means restriction education when provided during the ED presentation, with up to 100% of parents engaging in means restriction 2–3 weeks after hospital discharge [6, 7, 8]. Furthermore, gatekeeper education training for parents has shown to improve parental confidence in keeping their adolescent child safe from future suicide crises [9, 10]. Family‐based therapy has also demonstrated a reduction in suicidal ideation in suicidal adolescents [11, 12].

Concerningly, however, parents report feeling unsupported by the ED staff and processes and ill equipped to provide care to their adolescent child after they are discharged from the hospital [13, 14]. The period after ED discharge is often one of great concern for parents who subsequently undertake profound lifestyle alterations, such as leaving or reducing employment and abstaining from social interactions and events to provide constant monitoring and support to their child, with significant consequences for the parent and the family unit [4]. Also, there are often long delays between discharge and when the adolescent is able to connect with community‐based mental health services [15], placing increased pressure on parents to cope and navigate this turbulent time. Providing parents more support and evidence‐based information about to how care for their adolescent child and themselves may reduce the likelihood of repeat suicide crises and improve engagement with community programs [16].

Despite the vital role parents play supporting their adolescent child after hospital discharge, there has been little research which examines what support needs parents have during the period surrounding the ED presentation. This paper is the first explore the support needs of parents with the meaningful inclusion of people with lived experience throughout the research process and the writing of this paper. As such, this qualitative study sought to investigate what supports parents require when they accompany their suicidal adolescent, both during and immediately following the ED visit.

## 2 Methods

### 2.1 Study Setting

This qualitative study was approved by University of New South Wales Human Research Ethics Committee (HC220756). Written consent was obtained from all participants prior to data collection. This study draws upon data collected as part of a larger study which sought to examine two domains, first parents' experience of accompanying their adolescent child, who was experiencing suicide crisis, to the ED, which has been reported elsewhere [14], and second, the support needs of parents during and following the ED visit, which are reported here.

### 2.2 Recruitment

Twenty participants were recruited from the Australian community between January and March 2023, via Black Dog Institute's social media channels (Meta, X and LinkedIn). After engaging with the social media posts, potential participants were redirected to the study website, which provided study details and prompted them to complete the participant information sheet before completing a screening questionnaire to assess eligibility. To be eligible, participants must be a biological, step, or adoptive parent who had accompanied their adolescent child (12–18 years) to an Australian ED in the previous 5 years (i.e., since January 2018) for suicide crisis (ideation, feelings, plans or attempts). There were no exclusion criteria. A 5‐year timeframe was utilised to account for the changes in the ED processes in Australia during the COVID‐19 pandemic. Eligible participants completed the consent form prior to registering for the study. Participation was voluntary and participants were free to withdraw from the study at any time prior to data analysis. Based on the notion of information power, in which fewer participants are needed in instances where the sample holds a robust degree of information [17], a sample of 20–25 people was considered sufficient to ensure high information power given the diversity of the sample and specificity of the area of investigation.

### 2.3 Data Collection

The interview guide was developed in consultation with six peer researchers (including S.O.A., S.A.P. and J.L.) who are parents with a lived experience of accompanying their suicidal adolescent to the ED. Data were collected via online, semi‐structured, one‐on‐one interviews between February and May 2023. Interviews were conducted via a secure videoconferencing platform by the lead author (D.R.) and three peer researchers (S.O.A., S.A.P. and J.L.) who had been trained and supported to conduct the interviews. During the interviews, participants were asked to speak about their experience of their most recent ED presentation but were encouraged to draw on their previous visits if helpful when describing the most recent presentation. Interviews were digitally recorded and were reviewed by DR at the completion of each interview to assess for consistency of interview delivery. Recordings were de‐identified before being sent to a third‐party service for transcription.

### 2.4 Analysis

Due to the nature of interview, support needs were raised by participants throughout all sections of the interview, however, the interview guide also included a question regarding the support the participants would have liked to receive: ‘what support options would you have liked to be provided for yourself during the ED visit?’. All participant responses which pertained to desired support were included in the analysis.

Data was analysed following the six steps of reflexive thematic analysis [18], guided by the hermeneutics of empathy [19], and was led by the lead author (D.R.). First, in‐depth familiarisation with the interview transcripts was conducted which involved full immersion with the data corpus and repeated reading and reflecting on the transcripts. During this step, interviewers reviewed the transcripts of the interviews they conducted to produce one‐page summaries before discussing the shared ideas across the interviews as a group. Next, transcripts were read line‐by‐line and initial codes developed by the lead author who has extensive experience conducting qualitative analysis in suicide prevention research. These codes were then reviewed and discussed with the research team (D.R., F.S., K.B. and L.M.) to determine the final set of codes. At this phase, the codes relating to the support parents wanted during the ED presentation were removed from the larger dataset. These support data were then analysed separately, and codes were refined or expanded upon where appropriate. These codes were used as building blocks and sorted into ‘clusters of meaning’ [20] to then produce a set of three themes that were developed by the research team. These themes acted as central organising concepts to assemble the types of support parents wanted during this critical time.

### 2.5 Reflexivity Statement

Rigour was maintained throughout the analysis process through continued reflexivity, utilising the whole team during analysis, and maintenance of a detailed audit trail. The research team was comprised of academic researchers (including clinical psychologists), and peer researchers who brought unique professional and experiential wisdom which informed both data collection and interpretation. In alignment with the reflexive thematic analysis approach, the researched team engaged in reflection throughout the data collection and analysis. The experiential wisdom provided by the peer researchers supported our understanding of the participants experiences and ensured that important aspects of the ED journey were not misunderstood or excluded from analysis. Interviewers were encouraged to reflect on the interview process and to consider how their own life experiences and biases may have informed the data collection and interpretation. The lead author also maintained a reflection journal which included notes of interpretations, positionality and prior knowledge and how that may have impacted the analytic decisions throughout the research.

## 3 Results

### 3.1 Participant Characteristics

Twenty biological parents with a mean age of 46.9 years (SD = 4.3, range 40–55 years) completed interviews. Most participants were female (*n* = 18, 90%), roughly half were employed full time (*n* = 11, 55%) and 75% (*n* = 15) were married. The average age of the adolescent at the time of presentation was 15.35 years (SD = 1.79, range: 11–18 years), most adolescents were identified as female (*n* = 12, 60%), 25% as male (*n* = 5), 10% non‐binary (*n* = 2) and 5% as gender fluid (*n* = 1).

Three quarters (*n* = 15) of the presentations were for a suicide attempt, the remaining presentations were for suicidal thoughts without attempt (*n* = 5). Of those who had made a suicide attempt, most attempts were by overdose of medication (*n* = 12, 75%), with the remaining attempting via intentional self‐injury (*n* = 3, 25%). For half of the participants (*n* = 10) this was their first presentation to the ED for their adolescent childs' suicide crisis, the remaining participants had presented on two or more occasions (*n* = 10). Participants' most recent ED presentation for suicide crisis occurred between early 2018 and March 2023. To maintain participant anonymity information about the hospital attended was not collected. A more detailed exploration into the participants' most recent ED experience is reported by Rheinberger et al. [14].

### 3.2 Thematic Analysis

Reflexive thematic analysis resulted in identification of three themes outlining the types of support parents wanted as a result of the ED presentation: (1) Information to reduce the uncertainty of the ED presentation, (2) strategies to keep their adolescent child safe and support recovery, and (3) support for parent wellbeing. These findings were consistent across parents of both younger and older adolescents. A breakdown of each theme and related codes as well as exemplar quotes from participants can be found in Table 1. The age of the adolescent at the time of the ED presentation are also noted alongside each quote.

**Table 1:** Outline of themes structure with participant quotes.

Theme	Code	Quote
Involvement in ED care	What to expect during hospital presentation	Quote 1: The lack of information was difficult. Not knowing what they were gonna do, feeling that I had to go and harass them to ask anything what the next steps were or was it normal that she was vomiting so much? Can we give her anything? — Participant 16 (adolescent age: 14 years)
		Quote 2: I think more information about, you know, this is why and this is what it'll look like and this is why we think it's the best decision. — Participant 11 (adolescent age: 16 years)
		Quote 3: They could tell me they're doing this and this and this. This is the protocol and then this will happen. And then we understand it's not just physical, it's mental health. So, we're gonna do this and this and this, and this is what it could look like. They could have just shone a torch in this darkness, this place where I was at and saying, okay, this is where we are. This is where we're going. This is what we want. And that knowledge would've been wonderful to me. Cause I was just panicking. — Participant 3 (adolescent age: 17 years)
	An opportunity to discuss adolescents' crisis with staff	Quote 4: [The parent perspective is] relevant information. It's relevant. … Actually, being heard as the carer as well, what your genuine concerns actually are. When we all know our young people the best … and we are just not listened to … and don't have the capacity to fight the system to be heard. — Participant 18 (adolescent age: 14 years)
Information to support adolescent	What is necessary after discharge	Quote 5: If they could get something in the waiting room … kind of fundamental basic things that you need to do or watch for or you know, ask to keep someone safe. … people are so discombobulated … and so absolutely they should leave with, you know, information on you know, what triggers are, information on how things might get maintained, what to look for, and how to kind of respond. — Participant 20 (adolescent age: 15 years)
		Quote 6: I wish I knew then what I know now in terms of how better to have supported her at that time and afterwards, like we did a good job. I think, you know, we were, we, we, we were calling psychologists, we were talking to Amanda, we kept her out of school for a couple of days. You know, we just, we, we hugged her, we supported her, you know, we, but that only does, does so much and it doesn't get to the heart of what whatever that problem was. — Participant 7 (adolescent age: 14 years)
		Quote 7: I would expect that [during] that assessment at the ED, the person would walk away with a safety plan and that I, as a caregiver would know what's in that safety plan and why it's in there and what to do, like what are the agreed actions. — Participant 19 (adolescent age: 17 years)
		Quote 8: it's all very well and good to say, here's a phone number to call, but without knowing when you should call it, under what circumstances, what's going to happen when you call it? When you call this number, who are you going to talk to and … what are the options that they're going to be able to do to support you? — Participant 19 (adolescent age: 17 years)
	How to access support services	Quote 9: You're not going to fix them that very next day, but you can at least put in motion a very, like, high frequency process to … an expedited process to help them get the help that they obviously need. — Participant 7 (adolescent age: 14 years)
		Quote 10: I mean, young peer workers are phenomenal and having them working in with, you know, your young people who are in that crisis would be amazing … So having somebody else, … that gets them without having to explain everything and hold space for them would allow them that opportunity to breathe. — Participant 18 (adolescent age: 14 years)
		Quote 11: I think it would be amazing if parents in crisis like this were actually given, like a like an advocate, like a social worker case worker to kind of help them navigate the system. What's, what's actually available, what other options are there what other programs might help you know, what, you know, because someone who actually knows how to do it. — Participant 14 (adolescent age: 13 years)
	Education about suicide crises	Quote 12: I think that's really important that people actually have a much better understanding of suicide and how the experience of that person in that time … — Participant 19 (adolescent age: 17 years)
		Quote 13: So understanding, you know, what their mental, you know what normal mental health looks like, when should you be concerned — Participant 9 (adolescent age: 15 years)
Tools to support parent wellbeing	Where parents can get psychological support	Quote 14: What would have been helpful is if they said to me … ‘This situation's very hard for you and whether or not you feel that now we recommend you get to counselling. Here's a card of someone you can talk to.’ … Because if you are doing well, then you can look after the child better and these next few days [and] weeks are going to be really taxing. — Participant 3 (adolescent age: 16 years)
		Quote 15: I felt awkward as well. I think had the mental health team offered me something then I would've probably said like yes. … [My friend] was like, ‘oh, you should ask him to refer you to some something.’ But then I kind of just felt like, I felt awkward asking for that. — Participant 5 (adolescent age: 16 years)
	Recognition and compassion from ED staff	Quote 16: And there's that very real possibility that the carer is going to break down and start crying and say, ‘look, I'm not okay’. … So, it is being able to, to hold that carer in that moment … — Participant 18 (adolescent age: 14 years)
		Quote 17: I just tried to, you know, roll up my, my sweatshirt and kind of wedge it on my shoulder and tried to get like a, a little bit of sleep … no one really checked on us or checked on me to see if I needed anything. Like previously, like she'd been there with a suspected appendicitis and then, you know, the nurses was really good like, cause it was like really cold and they were coming back with some forwards and they were like ‘do you want a blanket or can we get you anything?’ But no, this was just like, we were just kind of left. — Participant 5 (adolescent age: 16 years)
	Tips for parent wellbeing	Quote 18: … you are allowed to want to vent, you are allowed to be angry. You're allowed to be frustrated. You're allowed to be sad. You're allowed to have all of those feelings … What we need is waiting rooms with brochures about, these are 10 things that you can do more often that will help make you happier. You are a complicated indoor plant. You need this amount of sunshine and you need that amount of good food. — Participant 1 (adolescent age: 16 years)
		Quote 19: Just the biggest thing was just to know that I wasn't the only person going through this and speak to other people with a lived experience of it that could provide some tips, advice, coping strategies you know, the tips like if you don't feel safe for yourself or your young person … putting you in contact with, with supports that could help, that would help you regain some of that power, some of that certainty. —rrParticipant 15 (adolescent age: 16 years)

*Note:* Adolescent age refers to adolescent's age at presentation to the ED. Adolescent names are replaced with pseudonyms.

#### 3.2.1 Involvement in ED Care

Parents were often not informed about the care their adolescent child was receiving but would have liked to receive more information about the hospital presentation, including what they were to expect in terms of treatment and outcomes, as well as an opportunity to discuss their adolescent child's crisis with staff.

##### 3.2.1.1 What to Expect During Hospital Presentation

Parents face considerable uncertainty during the ED visit, both regarding their child's wellbeing and the care provided. Parents were rarely updated about what the ED staff were doing to address the suicide crisis, or how they were treating medical issues which arose due to the adolescent's suicide attempt. Participants reported wanting to be provided information about what their adolescent child's care would look like during their time in the ED and, if necessary, their inpatient admission (see Quote 1, Table 1). This included being told what treatment options there were as well as the outcomes of interventions, such as those to reduce lethality of an overdose. Parents also wanted to be told why staff were using a specific treatment and what the pros and cons were of the approach. Parents also wanted more information about the ED process, such as information about what to expect after presenting, expected waiting times, who they could expect to speak with about their adolescent child's wellbeing, and the general flow of the presentation from triage through to admittance to a hospital ward.

Some parents spoke of having to repeatedly badger staff for more information about what to expect next but also to quell concerns that their adolescent child's symptoms were normal and not an indication of further deterioration. Parents felt that being provided any information about their adolescent childs' care would have reduced feelings of uncertainty and fear that staff were not responding to the suicide with the necessary urgency (see Quote 3).

##### 3.2.1.2 An Opportunity to Discuss Adolescents' Crisis With Staff

Parents felt that ED staff did not recognise and appropriately leverage their knowledge of their adolescent child's mental state. Many parents discussed being ignored by hospital staff, leading them to feel as though the staff did not care about ensuring the best result for the adolescent. Parents wanted ED staff to provide them an opportunity to discuss the context and nature of their adolescent child's suicide crisis, as they felt this would improve outcomes for their child and reduce the time spent in the ED. Discussing adolescent's crisis with staff would also have given parents a sense of control, which parents typically do not have once their adolescent child is in the care of the ED staff (see Quote 4). Participants also highlighted the importance of being able to have this conversation with staff confidentially, away from the adolescent, due to fears that overhearing the conversation would increase their adolescent childs' distress.

#### 3.2.2 Information to Support Adolescent

Parents would like more information about how to provide the care their adolescent child needs once they are discharged from the ED or hospital. This includes general information about how to keep them safe, information about accessing psychological support services in the community, where to find other, non‐clinical, support for their adolescent child, and education about mental health and suicide.

##### 3.2.2.1 What Is Necessary After Discharge

Participants shared that they felt they were not provided sufficient information about how to care for their adolescent child once they were discharged home from the ED or the hospital. Parents discussed wanting a recovery ‘roadmap’, so they felt confident to proceed with their adolescent child's care after discharge, which they anticipated would have alleviated the stress and fear the parents felt (see Quotes 5 and 6). Some participants mentioned the benefit of being provided medication for their adolescent child to help manage the immediate distress.

Parents wanted information to help them recognise warning signs which may be indicative of future suicide crisis and how to appropriately respond to these warning signs. Similarly, parents wanted to know what was included in their adolescent child's safety plans and preferred to be involved in the generation of the safety plan, so that they understood what was required of them, and how to help their adolescent child access the necessary support (see Quote 7). While many parents spoke of receiving a list of crisis helplines (e.g., Lifeline), they found they were not helpful. More information about the circumstances under which the helplines should be utilised would have improved their helpfulness (see Quote 8).

Parents wanted support information provided verbally by hospital staff so they had the opportunity to ask clarifying questions, but also wanted it provided on printed material which they could refer to later when they were not in distress.

##### 3.2.2.2 How to Access Psychological Support Services?

Parents often left the ED without a clear understanding how to access psychological support for their adolescent child but knew that psychological support was vital to reduce the psychological distress underpinning their adolescent child's suicide crisis. For some parents this was not their first presentation to the ED and many parents had faced challenges accessing support for their adolescent child, such as long waiting lists and financial difficulties paying for psychological support even if the sessions were subsidised under a mental health care plan, between visits to the ED or after an ED presentation. Participants reported wanting information about how to navigate the mental healthcare system and for the hospital to arrange appointments with mental health clinicians in the community or provide them with a referral (see Quote 9). Parents also spoke of wanting more information about how to access peer support workers or social workers (see Quotes 10 and 11) for their adolescent child, as alternatives or additions to more traditional psychological support services.

##### 3.2.2.3 Information About Suicide Crisis

Parents discussed wanting more information about suicide crisis, so they were in the best possible place to care for their adolescent child (see Quote 12). One parent noted the benefit of engaging with suicide specific training, such as Applied Suicide Intervention Skills Training (ASIST), so that parents had the skills and confidence to have conversations about suicide with their child. Other parents mentioned the benefit of more generalised mental health education so that they could more easily spot warning signs of declining mental health (see Quote 13), which they felt led to their adolescent child's suicide crisis.

#### 3.2.3 Tools to Support Parent Wellbeing

Participants recognised the value in making sure they were mentally well so they could provide the best care for their adolescent child. To address this, parents wanted information about how to access psychological support for themselves, tips for caring for themselves, and the power of informal support such as recognition and compassion from hospital staff.

##### 3.2.3.1 Where Parents Can Get Psychological Support

Many parents experienced significant mental health impacts due to the adolescent's suicide crisis and felt that this was impacting their ability to care for their adolescent child. Participants highlighted the importance of support to access their own psychological support, however parents reported they felt uncomfortable asking for such support from ED staff (see Quote 15). Parents discussed wanting the ED staff to suggest support that they could access as this would facilitate them accessing care without the perceived judgement or fear of being negatively perceived by staff. Parents were open to many different forms of support including trained therapists and psychiatrists, social workers and peer workers.

##### 3.2.3.2 Recognition and Compassion From ED Staff

Parents felt informal support in the form of recognition of their situation and compassion from ED staff would have improved their wellbeing during this time. Parents wanted staff to reassure them that they were doing a good job caring for their child, that they weren't to blame for the suicide crisis as well as acknowledgement that this was a challenging situation. Parents also spoke of the value of small acts of compassion, such as offering the parent a pillow or a blanket, or showing them where they can make themselves a warm drink during the long wait (see Quote 17). Parents felt such displays of compassion and verbal recognition of the struggles they were experiencing from the staff in the ED would go a long way in helping their psychological wellbeing during this time.

##### 3.2.3.3 Tips for Parent Wellbeing

As discussed, parents' wellbeing was significantly impacted during the adolescent suicide crisis, and parents wanted information about simple but impactful tips which could help. This included things to improve their mood such as reminders to spend time outside in the sunshine, to eat a balanced meal, to practice small acts of self‐care, or permission to feel the negative emotions (see Quote 18). Parents also wanted simple ways that they could connect with other parents going through this experience so that they felt less alone, and in company of people they felt they could openly share their feelings with without fear of judgement (see Quote 19).

## 4 Discussion

Parents provide vital support and care, beyond the scope of usual parenting, to their adolescent child during and following an ED presentation for suicide crisis. However, parents are rarely given sufficient support to provide this unique care. This study aimed to understand what types of support parents need when they attend the ED with their adolescent child who is experiencing a suicide crisis, so parents can best support the adolescent and reduce their subsequent risk. Qualitative analysis identified three domains of support which parents desired: (1) involvement in the ED care, (2) information about how to keep their adolescent child safe, and support their recovery, and (3) tools to support parents' wellbeing.

Providing information about the adolescent's treatment and care during the ED visit, delivered with care and at the right time, is one simple way to support parents during the ED visit. Studies exploring parents of ill adolescents and children found explaining hospital treatment processes was beneficial for parent and child outcomes [21, 22]. Keeping individuals abreast of treatment processes and providing concrete prognosis goals has been found to reduce distress experienced by parents of children and adolescents with cancer [23]. Given the high degree of psychological distress experienced by parents of adolescents experiencing a suicide crisis [4], it is important to maximise simple, effective support mechanisms to improve outcomes for parents. Incorporating elements of caregiver engagement when treating suicidal adolescents, as is seen with parents of other ill children [24], into hospital employee training and procedures may effectively improve parent confidence and skills in providing care after discharge [25, 26].

Parents in this study frequently identified wanting to be provided with more information about how to access psychological support for their adolescent child after being discharged from the ED or hospital. Parents report being overwhelmed and struggling to understand how to navigate the complex mental health systems [13, 14, 27]. Patients are often impacted by difficulties accessing services because of insufficient availability and long waiting lists [28, 29]. Furthermore, community mental health services targeted towards adolescents often have high entry thresholds, meaning in many instances, adolescents are only able to utilise services after they attempted suicide [15]. ED staff have reported local services are complex, with numerous service offerings (which frequently change), making it difficult for them to know what services they should be recommending for individuals experiencing self‐harm or suicide crisis [30]. As such, ED staff may not be well‐equipped to provide effective discharge care plans for this group. They may also lack the time and knowledge to develop care plans collaboratively with the young person and their parent or caregiver [31, 32]. Furthermore, adolescents do not often speak with a mental health clinician in the ED [33] and general ED staff are unlikely to have training or knowledge of mental health or suicide specific service options [30, 32]. Therefore, resources which help ED staff and help‐seekers identify and contact psychological services may help increase the uptake of such services [34], and effective methods of providing such information should be investigated. This is likely to have a profound impact through increasing parent confidence in ED staff [35], improve adolescent access to appropriate resources [36], and empower hospital staff to confidently provide care thereby reducing the feelings of futility experienced by staff [37].

Parents wanted information to help them with their own wellbeing so that they are able to provide the best possible care to their adolescent child. Parents' declining mental health surrounding an adolescent's suicide crisis is frequently noted in the literature, particularly increased anxiety, depression and fear [4, 13]. Anxiety and depression have been linked with decreased cognitive functioning [38], which may impact a parent's ability to make the best decisions about their adolescent child's care. Parents in this study highlighted the importance of receiving support for themselves throughout this journey, starting with compassion from ED staff, through to accessing psychological support in the community. Small acts of kindness from hospital staff, such as compassion and recognition of their feelings, helped individuals feel supported and gave them hope they could get through difficult periods [39]. However, parents in this study were unlikely to ask for help themselves for fear of judgement, and healthcare professionals have also suggested that they should offer support due to parents likely feeling guilty for wanting to care for themselves [13]. Worryingly, a lack of compassion from ED staff during the ED presentation is not only distressing for parents and their adolescent children but is likely to reduce later treatment engagement [40], which may increase their risk of future suicide crises. Parents in this study also noted the positive impact of simple tips to maintain their own wellbeing during this period. This suggests parents do not necessarily require in‐depth and voluminous support from hospital staff, but compassion such as offering cup of tea, recognition of their difficulties, and simple wellbeing strategies may have positive outcomes.

This study has several strengths; this novel investigation of parent needs during an ED presentation with their suicidal adolescent allows us to make an important contribution to the scant evidence surrounding parents' support needs. Next, the incorporation of lived experience expertise and voice in the development of the interview guide, data collection, 43 and analysis. Finally, the rigorous and detailed analysis process has resulted in greater clarity about parents' support needs. However, there are also a few limitations of note. First, most parents in this study were female, and the support needs of fathers may be different, particularly when considering the lower rates of mental health support service utilisation by males generally [41]. However, data indicate that mothers are more likely to engage in services for their adolescent child, particularly with respect to mental health concerns [42]. Second, the data included in this study were extracted from interviews, which encompassed multiple topics, and a more focused, in‐depth qualitative exploration of parent needs may uncover additional support requirements. That said, the sample size was substantial, and consistency across participant reports, and recent studies, for example, Cao et al., and Cafferty et al. [13, 27] suggests that the themes reported here are likely robust. Third, participants were recruited online, from across Australia, therefore it is possible that support needs may be different for those who do not have access to online resources. Fourth, participants' most recent ED presentation for suicide crisis were as far back as 5 years prior to the interview, therefore recollection of their needs during the ED presentation may be diminished. However, this may be countered by the added benefit of more time navigating the system after crisis, enabling participants to have a better understanding of what has been helpful since the visit and therefore noting that earlier access to such support could have been beneficial. Fifth, while the findings reported here are in alignment with other Australian studies, more research may be needed to help understand the support needs in other countries in light of differences in care provisions for adolescents experiencing suicide crises.

Together, these findings suggest that parents have a considerable appetite for information which empowers them to provide necessary care for their adolescent child, rather than wanting others, such as a healthcare professional, to completely take over this care. If empowered appropriately, parents may feel more confident to support their adolescent child once home in the community [43], thereby reducing the risk of future suicidal crises and relieving some of the pressure on the ED. Additionally, these findings provide a preliminary evidence base of what support parents require during the ED presentation, which could easily be incorporated into standard care delivery as they are in alignment with person‐centred care practices already prioritised in healthcare settings [44]. Despite this, few measures have been considered to address these support needs before now. Further research is needed to understand what support parents find the most helpful, and identify what gaps exist between the support parents need and what is currently being provided in ED settings. Such information, alongside these findings, could be further developed through co‐design practices to ensure that interventions or training are more aligned with parent needs [45]. Co‐designed interventions with parents of young people in high‐risk populations, such as trans youth or children with ADHD, have shown to effectively improve parent confidence supporting their children and improvements in parent wellbeing [46, 47]. Healthcare providers could also benefit from training on how some of the simple support needs, such as compassionate and informative interactions with parents, could be integrated into their care delivery to suicidal adolescents.

## 5 Conclusion

Parents require a variety of supports as a result of the ED presentation and their adolescent child's suicidal crisis. Parents identified the need to be informed and educated so they feel empowered to provide the care their adolescents require. Simple steps to improve parental wellbeing are extremely important to ensure parents are in the best possible position to provide the life‐preserving care their adolescent after discharge from the hospital. These findings provide a preliminary outline of the parental supports that could easily be integrated into the ED procedures and which resources may need to be developed to educate and support parents.

## Author Contributions

**Demee Rheinberger:** conceptualisation, investigation, methodology, validation, writing – review and editing, writing – original draft, project administration, formal analysis, data curation. **Katherine Boydell:** conceptualisation, writing – review and editing, methodology, supervision, validation. **Susanne Oliver Armstrong:** methodology, writing – review and editing, formal analysis, validation, investigation. **Sally Ann Pollard:** formal analysis, methodology, writing – review and editing, validation, investigation. **Julia Lessing:** validation, methodology, investigation, formal analysis, writing – review and editing. **Lauren McGillivray:** writing – review and editing, methodology, validation, conceptualisation, supervision. **Fiona Shand:** methodology, validation, conceptualisation, writing – review and editing, supervision.

## Ethics Statement

The study was conducted according to the guidelines of the Declaration of Helsinki and approved by the University of New South Wales Human Research Ethics Committee (HC220756). Written consent was obtained from all participants prior to data collection.

## Conflicts of Interest

The authors declare no conflicts of interest.

## Data Availability

The data that support the findings of this study are not publicly available due to ethical considerations.
